# Improving the Accuracy of Estimates of Indoor Distance Moved Using Deep Learning-Based Movement Status Recognition

**DOI:** 10.3390/s22010346

**Published:** 2022-01-04

**Authors:** Zhenjie Ma, Wenjun Zhang, Ke Shi

**Affiliations:** School of Computer Science and Technology, Huazhong University of Science and Technology, Wuhan 430074, China; mazhenjie@hust.edu.cn (Z.M.); zhangwenjun@hust.edu.cn (W.Z.)

**Keywords:** indoor positioning, movement distance estimation, movement status recognition

## Abstract

As a result of the development of wireless indoor positioning techniques such as WiFi, Bluetooth, and Ultra-wideband (UWB), the positioning traces of moving people or objects in indoor environments can be tracked and recorded, and the distances moved can be estimated from these data traces. These estimates are very useful in many applications such as workload statistics and optimized job allocation in the field of logistics. However, due to the uncertainties of the wireless signal and corresponding positioning errors, accurately estimating movement distance still faces challenges. To address this issue, this paper proposes a movement status recognition-based distance estimating method to improve the accuracy. We divide the positioning traces into segments and use an encoder–decoder deep learning-based model to determine the motion status of each segment. Then, the distances of these segments are calculated by different distance estimating methods based on their movement statuses. The experiments on the real positioning traces demonstrate the proposed method can precisely identify the movement status and significantly improve the distance estimating accuracy.

## 1. Introduction

The increasing market demand for indoor location-based services has driven a rapid development in indoor positioning techniques in recent years. A large number of indoor positioning solutions based on WiFi [1], Bluetooth [2], and UWB [3] have been proposed. After deploying these solutions in indoor environments, the positioning traces of moving people or objects can be recorded, and the distances traveled by these moving people or objects can be estimated. Indoor distance estimation is a frequently used function in many real applications, such as workload statistics and job allocation in warehouse logistics, and is highly useful for optimizing workflow and improving productivity.

However, due to the uncertainties of wireless signal propagation and corresponding positioning errors, accurately estimating movement distance from positioning traces still faces challenges. In comparison with outdoor environments, indoor environments are more challenging for positioning. The ambient objects in the indoor environments, such as walls, the ground, furniture, and the human body, commonly lead to Non-Line-of-Sight (NLOS) propagation. These objects also may cause the wireless signals to reflect, scatter, and diffract. Compared with outdoor environments, this causes more severe multipath effects and more greater distance error [3]. 

Regarding localization, the indoor environment is more dynamic than the outdoor environment. Human movement, opening and closing of doors, and changes in the location of furniture may affect signal propagation. Therefore, the signal received is highly time-varying at a fixed position, which results in significant positioning error [1].

Numerous methods have been proposed to reduce the positioning error; for example, WiFi fingerprinting-based methods can limit the error to the meter level [4,5,6], and UWB Time Difference of Arrival (TDOA)-based methods [7] can limit the error to the sub-meter level. However, deviations between the returned positioning results and the actual positions exist. Directly calculating the distance via linear interpolation may lead to a significant error; for example, even when the object is stationary, the position returned by the positioning system at each positioning time interval varies, which causes direct calculations to significantly overestimate the distance.

Some data smoothing methods [8] can be utilized to process the positioning trace data, remove outlier data, ensure the processed trace is closer to the actual trajectory, and improve distance estimation accuracy. These methods work well only when the object’s movement falls into a smooth pattern without frequent and sudden changes; for example, when a worker in the warehouse walks from one pick-up location to another, his movement is usually smooth.

However, when a worker in the warehouse operates in a narrow corridor between shelves to check and sort items, his movement falls into another pattern that involves a large number of sudden changes in direction and speed. The data smoothing methods may not work well under this pattern, and some customized data processed methods are needed. 

When a person walks around an indoor space, his trajectory usually consists of several segments, and each segment corresponds to a movement pattern; for example, when a worker works in a warehouse, some parts of his trajectory are smooth, some are characterized by a large number of changes, and some are stationary. The estimating method needs to handle all of these statuses well. To the best of our knowledge, there is no single data smoothing method that can meet this requirement.

Some outdoor positioning and navigating methods have been proposed to estimate the distance/time traveled using low rate and error-prone Global Positioning System (GPS) raw data [9]. These methods either utilize road network information to reconstruct the trajectory and make the reconstructed trajectory conform to the actual trajectory [10], or utilize extra sensors, such as an accelerator and compass, to calibrate the GPS points [11]. However, apparent road networks, which constrain the target’s movement in indoor space, may not exist. Moreover, extra sensors may incur additional costs.

This paper proposes a movement status recognition-based distance estimating method to improve the estimation accuracy. We divide the positioning traces into segments and use a deep learning model to determine the motion status of each segment. Then, the distances of these segments are calculated by different distance estimating methods based on their movement status. 

The main contributions of this paper can be summarized as follows. First, we split the recorded trace into segments and propose an encoder–decoder deep learning-based model to determine the motion status of each segment. Second, different data smoothing methods are applied for each segment to process the raw data points and reconstruct the trace based on its motion status. Additionally, the experiments on the real positioning traces demonstrate the proposed method can precisely identify the movement status and significantly improve the distance estimating accuracy.

The remainder of this paper is organized as follows. Section 2 discusses related work, and our movement status recognition-based distance estimating method is presented in Section 3. Section 4 presents the performance evaluation details. Finally, Section 5 concludes the paper and notes the direction of future work.

## 2. Related Work

### 2.1. Distance Estimation

As a result of the development of wireless and sensing technology, more indoor positioning systems have been deployed to obtain the position traces of moving objects in indoor space. Although some systems utilize more complex signal characteristics, such as channel state information, to improve positioning accuracy [12], an error at each location point still exists due to vulnerable signal transmission. Due to this error, the recorded trace deviates from the actual trajectory and results in a significant cumulative error in distance estimation. 

Data smoothing and filtering techniques, such as moving average filtering and Kalman filtering, can be used to eliminate outliers from traces to make the recorded trajectory closer to the real trajectory [8]. As statistical methods, data smoothing and filtering reduce wide variances or statistical noise to detect trends. For a trace following a simple pattern, these approaches work well. However, a trace usually contains multiple patterns [13]. Thus, it is hard to derive a single data smoothing and filtering method that can achieve better performance.

In outdoor positioning and navigation systems, researchers have presented methods to accurately estimate the distance from GPS traces. Brunsdon et al. [14] presented a method based on the idea of the principal curve to reconstruct the trajectories from GPS point data. Map matching-based methods [15] have been proposed to improve the accuracy of the reconstructed trajectory by utilizing the information of underlying road networks. Mizzi et al. [16] proposed a method to identify the pedestrian mobility characteristics on a road network to help reconstruct trajectories. Lopez et al. [17] studied the effect of missing GPS observations on the traveled distance estimation and proposed a regression model. Dewhirst et al. [11] proposed a method combining accelerometer-based speed and magnetometer heading estimates (dead reckoning) with low fix rate GPS drift correction to improve the accuracy of path and distance traveled estimates for studies of animal locomotion. 

Prentow et al. [18] proposed a method to estimate and iteratively refine an underlying route network from a large positioning trace set in indoor space. Then, the trace is aggregated and cleaned, which facilitates distance estimation. However, the constructed route networks may not be as accurate as road networks.

### 2.2. Movement Status Recognition

Movement status recognition can be considered as time series classification (TSC). Many TSC methods [19] have been proposed, most of which can be divided into three types: distance based, feature based, and ensemble based. Here, time series data are positioning traces (which some researchers call trajectories). Some existing trajectory classification methods aim to differentiate between trajectories (or segments) of different statuses, such as motions, transportation modes, and human activities [20,21].

TraClass [22] generates a hierarchy of features by partitioning trajectories and exploring region-based and trajectory-based clustering and assigns class labels to moving objects based on generated discriminative features. Joo et al. [23] utilize a Hidden Markov Model (HMM) to classify the trajectories into various kinds of foragers’ motion patterns. Gao et al. [24] use a Minimum Bounding Rectangle (MBR) to represent trajectory data and use the k Nearest Neighbor (kNN) approach to classify the trajectory. Zheng et al. [13] constructed a decision tree-based classifier to divide a user’s trajectory by transportation modes.

Most existing methods extract features from each trajectory and build a model to classify each trajectory. For example, velocity and velocity change rate can be used to distinguish stationary, walking, and driving statuses. However, it is hard to identify the universal and identifiable features for all application scenarios due to the motion uncertainty and noisy trace data. 

Due to the rapid development of artificial intelligence, deep learning-based TSC methods, such as convolutional neural network (CNN)-based methods, have rapidly emerged and achieved a competitive level of performance [25,26]. Additionally, deep learning-based methods allow joint end-to-end feature extraction and classification. 

Liu et al. classify travel modes of raw human trajectories using a bi-LSTM neural network [27]. They further utilize a gated recurrent unit (GRU) [28] to model spatial-temporal correlations of the trajectories to improve classification accuracy. A CNN architecture [29] is also proposed to infer transportation modes from GPS trajectories. Unlike these methods using CNN or RNN to extract high-level features, we present a customized encoder–decoder architecture that can extract features and remove the positioning errors simultaneously to improve the accuracy.

## 3. Methodology

### 3.1. Overview

**Figure 1:**
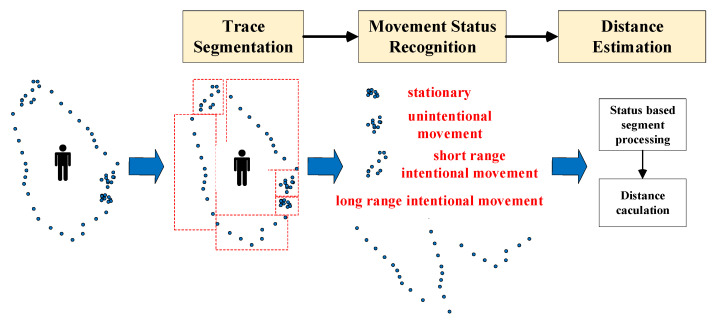
Overview of our method.

Figure 1 shows the overview of our proposed approaches, comprising three main steps: trace segmentation, movement status recognition, and distance estimation.

A trace *P* recorded by a positioning system is represented by a series of chronologically order points (*p*_1_, *p*_2_, …, *p_n_*), where each point consists of a geospatial coordinate set and a time stamp, such as *p_i_* = (*x_i_*, *y_i_*, *z_i_*, *t_i_*). Given a trace *P* and a time interval [*t_i_*, *t_j_*], where *t_i_* < *t_j_*, the segment *P_s_* is defined as a subset of *P* such that *P_s_* contains all points between *t_i_* and *t_j_* of trace *P*. 

Trace segmentation divides a trace *P* into continuous segments with the same time interval for further classification. Suppose the sampling rate of the positioning system is fixed, and each segment contains the same number of points. To accurately classify the movement status, each segment should be homogeneous in its movement status characteristics and contain enough characteristics information to distinguish between different statuses. Therefore, the time interval value should be carefully selected based on application scenarios. We will study adaptive segmentation methods with flexible time interval settings in future work.

After segmentation, movement status recognition solves the problem of assigning segments to classes of similar movement status characteristics using a deep learning-based classifier. According to moving objects’ behavior and spatial-temporal change, the status is divided into four classes: long-range intentional movement, short-range intentional movement, unintentional movement, and stationary. 

The long-range intentional movement represents the motion mode when an object moves from one position to another to complete specific work; for example, a worker in a warehouse walks from one pick-up location to another. It usually causes a relatively significant and smooth spatial change in a given time interval. The short-range intentional movement represents the motion mode when an object moves around a position to complete specific work; for example, a worker in a warehouse walks around one pick-up location to check and sort items. In this mode, the spatial information changes rapidly and repeatedly in a relatively small range.

Stationary status means there is no spatial change in a given time interval. In real application scenarios, an entirely stationary status is rare; for example, a worker in a warehouse is hardly still even when he or she is resting. Under this circumstance, the worker may move slightly around his resting position. Unintentional movement is represented by this motion mode. Compared with stationary status, unintentional movement usually has more spatial change. Compared with short-range intentional movement, the motion caused by unintentional movement is more random.

After status recognition, each segment is processed by a data smoothing method decided by its status. Then the distance of each segment is calculated using processed data, and the total distance traveled is obtained by summing all these segments’ distance.

The details of the deep learning-based classifier and status-based distance estimation are given in the following two subsections.

### 3.2. Deep Learning-Based Movement Status Recognition

The motion characteristics of moving objects are uncertain. Moreover, it is hard to derive the error model of the positioning system due to vulnerable wireless signal transmission. Therefore, building an explicit model describing the relations between movement status and trace segment is an unsolved (and highly challenging) problem. 

Instead, we use an end-to-end deep learning network to build an implicit model by observing labeled examples. By observing the relationship between trace segments and ground truth movement status, a neural network can learn an implicit representation of the relationship. Then, it can use this representation to identify statuses of unlabeled trace segments.

**Figure 2:**
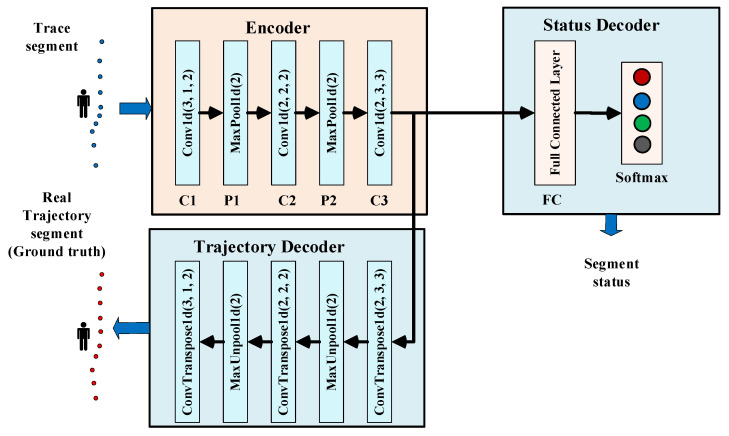
Classifier’s architecture: the classifier takes the trace segments as inputs and generates the status labels. For each Conv1d and ConvTranspose1d, the three values shown are [<kernel size>, <stride>, <padding>]. For each MaxPool1d and MaxUnpool1d, the value shown are [<kernel size>].

We devised a classifier for implementing movement status recognition, which takes the trace segments as inputs and generates the status labels as outputs. As shown in Figure 2, the proposed classifier adopts an encoder–decoder architecture, with two parallel decoders feeding off the encoder. 

The encoder takes in the input trace segment and generates a concise representation that feeds into the location and trajectory decoder. The encoder consists of a one-dimensional convolutional layer C1, a pooling layer P1, a one-dimensional convolutional layer C2, a pooling layer P2, and a one-dimensional convolutional layer C3. For C1 to C3, the kernel numbers are 64, 128, and 256. ReLU is used as the activation function. For P1 to P2, max pooling is used, and the subregions are nonoverlapping with a size of 2.

The trajectory decoder uses the concise representation generated by the encoder to reproduce the ground truth trajectory corresponding to the input trace segment by de-convolutional and de-pooling operations. For each one-dimensional convolutional layer of the encoder, there is a corresponding one-dimensional de-convolutional layer in the trajectory decoder. For each pooling layer of the encoder, there is a corresponding de-pooling layer in the trajectory decoder. Training the trajectory decoder to reproduce the actual trajectory can teach the encoder to remove the error caused by the positioning system. Recall that vulnerable wireless signal transmission causes random positioning errors in the input trace segments. If we do not correct these random errors, the classifier will not learn the movement status characteristics, thereby severely limiting its capability. To remove positioning errors, it needs training data that has ground truth trajectories as targets. The actual position data need to be gathered in the data collecting process.

The status decoder, which consists of a fully connected layer and a softmax layer, provides the classification results. The fully connected layer generates the final feature maps for classification. The softmax layer provides the posterior probability of each class which is coded in one-hot format and passed to the output.

Our encoder–decoder classifier takes trace segments as the inputs. The trajectory decoder generates the actual trajectories as outputs, and the status decoder generates the status classes as outputs. We employ L2 comparative loss for the trajectory decoder and cross-entropy loss for the status decoder. The overall loss function is a weighted sum of the losses of the trajectory decoder and the status decoder. 

We utilize labeled trace segments and ground truth trajectory segments to train the classifier end-to-end during the training phase. The classifier learns to remove the positioning errors utilizing the trajectory decoder. Because the trajectory loss and status loss are summed to update the encoder, the status decoder obtains access to the information regarding removing positioning errors, thus enabling it to learn and predict accurate movement status. Once the classifier is trained, the trajectory decoder is no longer needed. Only the encoder and status decoder are stored, and continuously receive the trace segments and identify their movement status.

### 3.3. Distance Estimation

After movement status recognition, each trace segment has a status label. The trace segments are processed, and the corresponding distances are calculated using specific methods decided by the segments’ labels.

For the trace segment labeled as stationary, the corresponding distance is zero. We use the Kalman filter to process the raw trace segment labeled as long-range intentional movement. The Kalman filter works in a two-step process: prediction and estimation. In the prediction step, the filter produces estimates of the current position along with their error probabilities. These estimates are updated using a weighted average of the raw position data with more weight being given to the estimates with higher certainty. The predicted position is compared with the actual poisoning data to obtain the optimum output in the estimation step. Therefore, noises and errors of the raw data are removed. The distance is calculated based on processed trace segments. 

The long-range intentional movement has a clear and stable motion pattern, for which the Kalman filter is suitable for making an accurate prediction. The processed trace segment is closer to the actual trajectory than the raw data segment. However, it is hard for the Kalman filter to accurately predict the trace segments labeled as short-range intentional movement and unintentional movement; in particular, it is hard to determine whether raw data spikes are caused by positioning errors or sudden motion changes. Hence, we use least-squares polynomial fitting to process the raw trace segments and generate a polynomial curve to approximately represent the actual trajectory. The length of the generated polynomial curve is the distance. 

Finally, the distance corresponding to the whole trace is the sum of the distances of all the trace segments.

## 4. Performance Evaluation

In this section, we verify the effectiveness of the proposed method based on the data obtained from the real environments and make comparisons with other existing methods.

### 4.1. Setup

**Figure 3:**
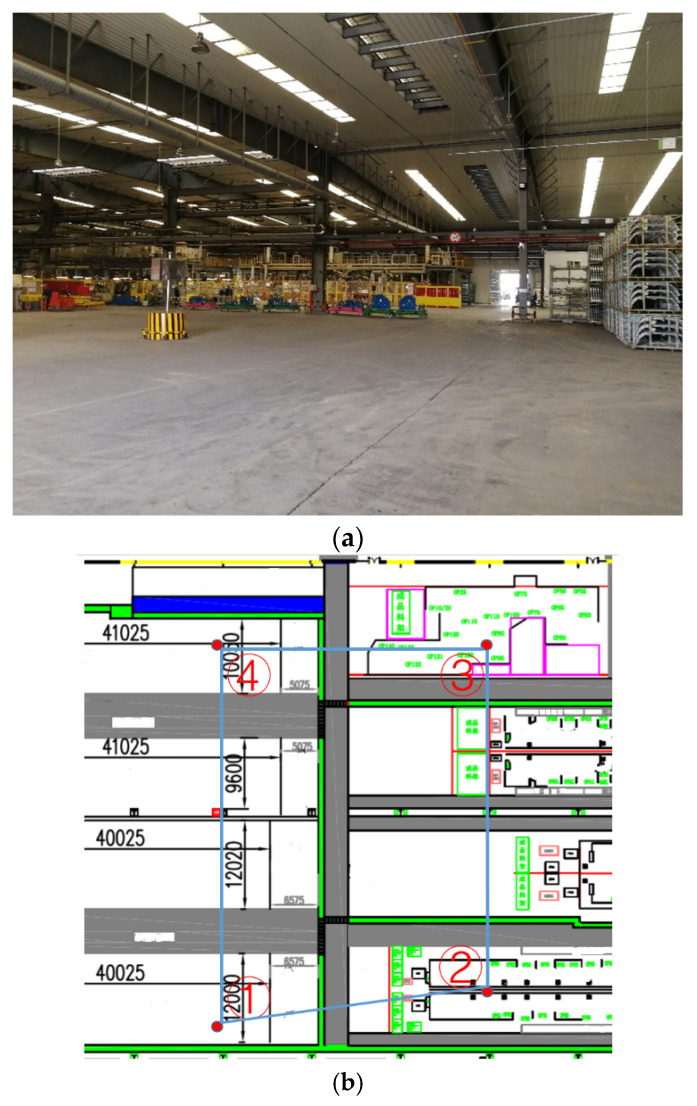
Experimental environment. (**a**) Auto parts factory space; (**b**) Space layout.

We collect the data from a TDOA-based UWB location system deployed in an auto parts factory space (see Figure 3a). As depicted in Figure 3b, four UWB positioning anchors (indicated by red dots) were deployed on the site. All the anchors utilize the network time protocol to implement full time synchronization. The system locates workers wearing UWB tags at a frequency of 2 Hz. The tag sends a broadcast message, and the anchor receives it and records the arrival time. Then, all the anchors send the arrival time of receiving this message to the location server. Because the location of anchors is different, the different anchors will record the different arrival times. The location server can calculate the location of the tag by a hyperbolic algorithm. The TDOA methodology enables the simultaneous tracking of multiple tags within the system. If there are enough computing and storage resources, our method can simultaneously process multiple users’ traces. 

**Figure 4:**
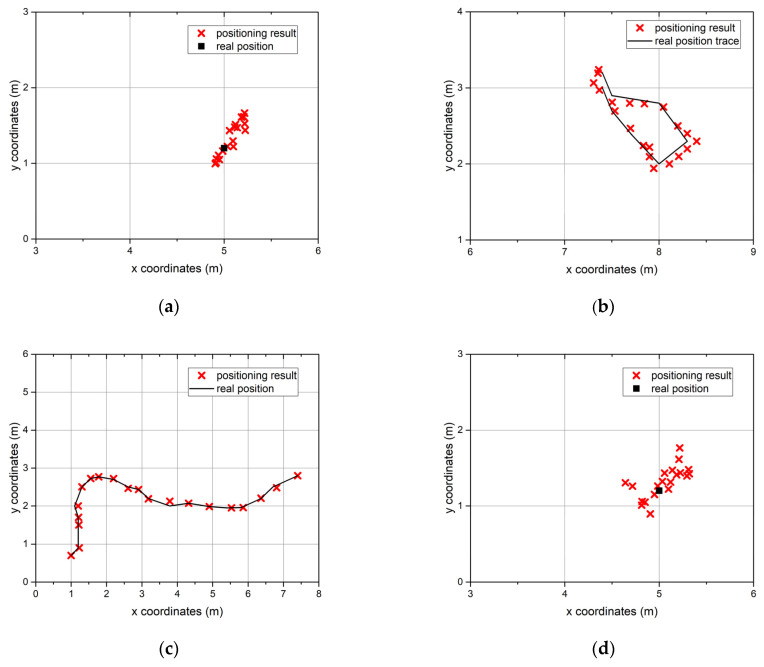
Ten-second trace samples of different movement statuses collected by the deployed UWB positioning system. (**a**) stationary; (**b**) short-range intentional movement; (**c**) long-range intentional movement; (**d**) unintentional movement.

We manually set 12 different paths that served as ground truth data. Each path consisted of pre-defined positioning points that can be further divided into sub-sequences corresponding to different movement statuses. The path length varied between 200 and 220 m. The average value was 210 m. The workers wearing the UWB tags walked along these paths 60 times. Each movement status lasted at least 25 s. Each path consisted of three segments corresponding to the long-range intentional movement status, two–three segments corresponding to the short-range movement status, three segments corresponding to the stationary status, and two–three segments corresponding to the unintentional movement status. The collected data contains 7468 trace segments. Figure 4 shows the trace samples of different movement statuses collected by the deployed UWB positioning system.

Experiments were conducted on a computer server with an Intel Xeon processor (2.30 GHz), 32 GB of memory, and Nvidia Tesla K80 12GB graphics card. 

### 4.2. Movement Status Classification Performance

#### 4.2.1. Metrics

We use the following three metrics to evaluate the performance of our classifier: precision (P), recall (R), and F1-score (F1). 

Precision is the fraction of true positive samples among the samples that the classifier labels as positive. Recall, also known as sensitivity, is the fraction of samples labeled as positive among the total number of positive samples. Precision is defined as:

(1) $$P=\frac{\sum_{i=1}^{m}TP_{i}}{\sum_{i=1}^{m}TP_{i}+\sum_{i=1}^{m}FP_{i}}$$

and recall is defined as:

(2) $$R=\frac{\sum_{i=1}^{m}TP_{i}}{\sum_{i=1}^{m}TP_{i}+\sum_{i=1}^{m}FN_{i}}$$

where *TP* is the number of true positives labeled by the classifier, *FP* is the number of false positives labeled by the classifier, *FN* is the number of false negatives labeled by the classifier, and *m* is the number of classes. Here, *m* is 4.

The F1-score is a means of combining the precision and recall of the classifier, and is defined as the harmonic mean of the classifier’s precision and recall:

(3) $$F1=\frac{2\left(P*R\right)}{P+R}$$

#### 4.2.2. Classifier Implementation and Training 

**Figure 5:**
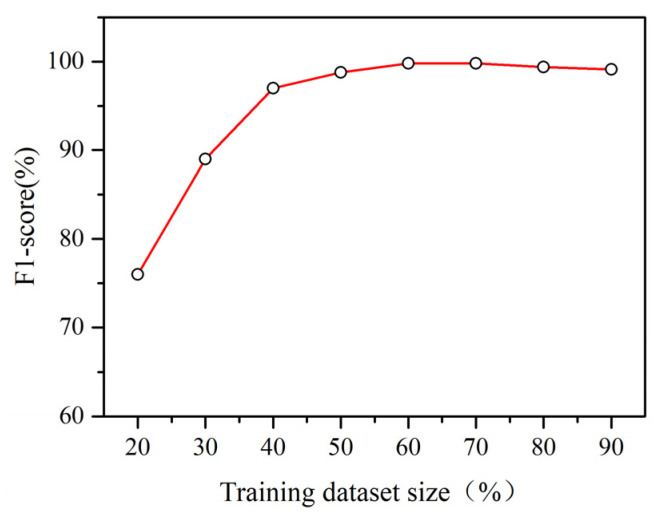
The impact of the training dataset ratio.

We implemented the proposed classifier in PyTorch. The recorded trace segments and corresponding paths’ sub-sequences were used to train this classifier. When training the model, we used Adam as our optimizer. Figure 5 shows the effect of the training dataset ratio on the performance of our classifier. 

We found 60% and 70% are the best training dataset ratios for achieving the best performance. When this ratio is low, there are few examples in the training dataset. This results in a low testing accuracy because the model overfits the training dataset or the training dataset is not sufficiently representative. When the percentage of data in the training dataset is higher than 70%, the accuracy is still high but slightly lower than the best value. Too many examples may cause the training dataset to be over-representative. Therefore, we allocated 60% of the available data for training, and the remaining 40% data were allocated to the test datasets. 

**Figure 6:**
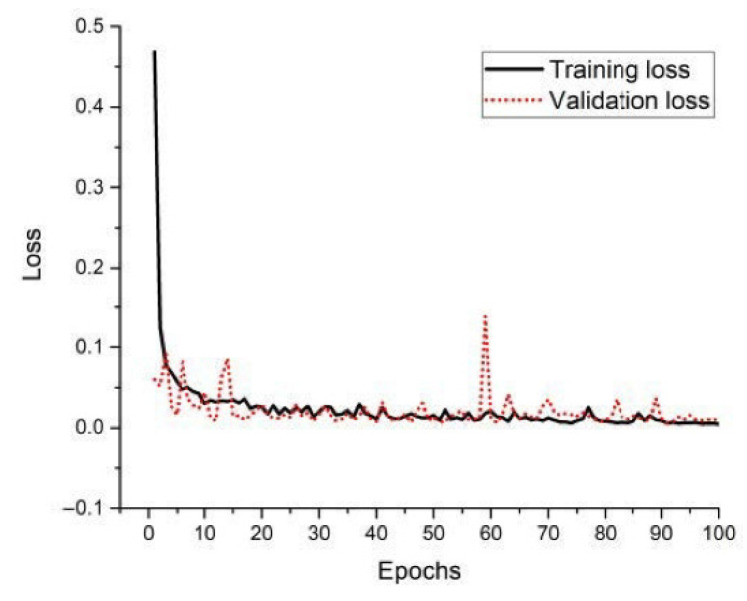
The loss during the training process.

Figure 6 plots the training and validation loss of our classifier during the training process. It is obvious that the training and validation loss both converge and have no significant change after 100 epochs. To limit training time, our training process terminated after 100 epochs.

#### 4.2.3. Comparison with Other Classifying Methods 

We compared our classifier with three methods: kNN, SVM, and CNN. Both kNN and SVM first extract the features from the raw trace segments, including the range of coordinate changes and its variance, the average velocity, and their variance, and then implement the classification based on these features. We found that directly feeding the noisy and error-containing data into the kNN or SVM classifier resulted in poor performance. In order to demonstrate the effectiveness of the trajectory decoder, the CNN classifier adopts a similar architecture to our classifier but does not contain the trajectory decoder.

The evaluation results are listed in Table 1. SVM performed slightly better than kNN because SVM maps the feature data to a higher-dimensional space and makes it easier to find a linear decision line in this new space. CNN performed slightly better than SVM and kNN. It shows that the end-to-end learning capacities provided by deep learning improve the classification accuracy without heavy crafting in data pre-processing compared with feature engineering. However, the precision and recall values of these three classifiers are all lower than 90%. Our classifier achieves remarkable performance and outperforms the other methods, and the precision and recall are above 97%. With the help of the trajectory decoder, the encoder can effectively remove the errors caused by the positioning systems.

**Figure 7:**
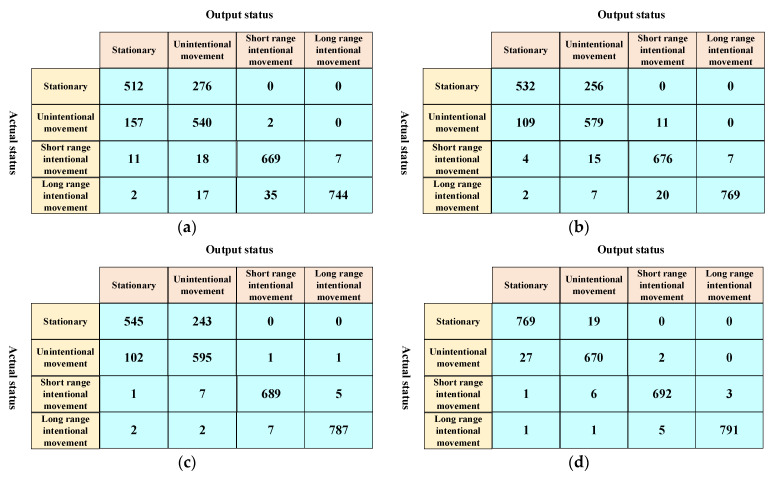
Confusion matrices of different classifiers. (**a**) kNN; (**b**) SVM; (**c**) CNN; (**d**) Ours.

In order to obtain a deeper insight into the performance, the confusion matrices of all four classifiers are given in Figure 7. We found the most challenging part was distinguishing stationary and intentional movement status. The misidentification rates of kNN and SVM are above 30%, and even for CNN, this value is close to 30%. The reason for this is that the difference between the unintentional movement and the stationary status is not significant, and the positioning errors easily blur this difference. By utilizing the trajectory decoder to restore the output of the encoder to the ground truth trajectory, the encoder of our classifier learns the ability to remove the positioning errors caused by vulnerable wireless transmissions, which significantly reduces the misidentification between these two statuses. It also results in more accurate identification of the other statuses by our classifier. 

Table 2 provides the execution time (including training time and inference time) of all four classifiers. For kNN, no training is needed, but inference (represented by average classification time for one segment) is high because it needs to compute the distance of each trace segment in the training dataset from the testing trace segment. Training an SVM classifier involves solving the quadratic problem and choosing the support vectors, which leads to a high training time of 6.3 s in our experiment. The inference time of SVM is lower than that of kNN because SVM only needs to determine the side of a hyperplane on which a given point lies. Training a deep learning-based model is computation intensive. As a result, the training time of CNN and our classifier is 451 and 876 s, respectively, which is much higher than that of SVM. Compared with CNN, our classifier needs almost twice the training time because our classifier introduces an extra trajectory decoder. In the testing stage, CNN and our classifier have the same architecture, which leads to the same inference time. The inference time is low because the convolution computation can be quickly undertaken by matrix computation.

### 4.3. Impact of Trace Segment Size

**Figure 8:**
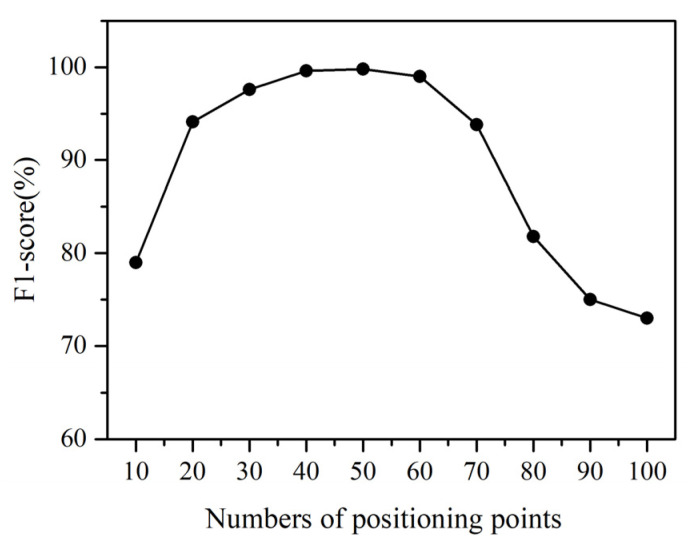
The impact of trace segment size.

Figure 8 shows the impact of trace segment size on our classifier’s accuracy. Because the sampling frequency is fixed, we use the number of positioning points in a trace segment to represent its size. When the size is small, a trace segment contains few positioning points and may not carry enough information for our classifier to successfully discriminate between the movement statuses. For example, when a trace segment contains 10 points, the corresponding movement lasts only 5 s because the sampling frequency is 2 Hz. It is hard for our classifier to distinguish between stationary status and unintentional movement status. It is also hard for our classifier to distinguish between long-range and short-range movement statuses. The F1-score drops below 0.8. 

As the number of positioning points grows, the trace segment is more sufficiently representative, which leads to a higher F1-score. Because one movement status lasts at least 25 s in our experiment, we find the best F1-Score when the number of points is 50. When the size is too large, a trace segment may not be homogeneous in its movement status. Some positioning points correspond to a given movement status, and some correspond to another, which has a significantly negative effect on the classification accuracy. This explains why the F1-score decreases when the number of points exceeds 50. The decreasing trend accelerates as the size increases further.

The results prove that the trace segment size has a significant effect on the final performance. Now the segmentation method prefers a small time interval to ensure the movement status of the divided segment is homogeneous. In real applications, the duration of a movement status varies. The small value of the time interval may cause a trajectory part corresponding to a single movement status to be divided into several segments. We will study adaptive segmentation methods that can divide the trace based on motion characteristics in future work.

### 4.4. Distance Estimation Results

#### 4.4.1. Metrics

We used mean absolute error (MAE) and mean absolute percentage error (MAPE) as the metrics to evaluate the performance of distance estimation. These two metrics are defined as:

(4) $$\mathrm{MAE}=\frac{\sum_{i=1}^{n}\left|y_{i}-\hat{y_{i}}\right|}{n}$$

(5) $$\mathrm{MAPE}=\frac{1}{n}\overset{n}{\underset{i=1}{\sum }}\frac{\left|y_{i}-\hat{y_{i}}\right|}{y_{i}}$$

where $y_{i}$ is the actual distance, $\hat{y_{i}}$ is the estimated distance, and *n* is the number of traces. MAE is the absolute value of the difference between the estimated value and the actual value. It gives less weight to outliers, which is not sensitive to outliers. One problem with MAE is that the relative size of the error is not always obvious. Sometimes it is hard to tell a large error from a small error. MAPE is the absolute error normalized over the actual value, computed for every data point and then averaged, which allows the error to be compared across data with different scales.

#### 4.4.2. Comparison with Other Distance Estimating Methods 

We compared the performance of our approach with the following methods:

1. ED, calculating the Euclidean distance between the consecutive points of raw trace and using the sum of these distances as the estimated distance;
2. KF, utilizing Kalman filter to process the raw trace, and using the distance of processed trace as the estimated distance;
3. LSF, utilizing least square fitting to process the raw trace, and using the distance of processed trace as the estimated distance;
4. kNN-S, dividing the raw trace into segments, classifying the statuses of segments by kNN, and using status-based estimation to obtain the final distance;
5. SVM-S, dividing the raw trace into segments, classifying the statuses of segments by SVM, and using status-based estimation to obtain the final distance;
6. CNN-S, dividing the raw trace into segments, classifying the statuses of segments by CNN, and using status-based estimation to obtain the final distance.

**Figure 9:**
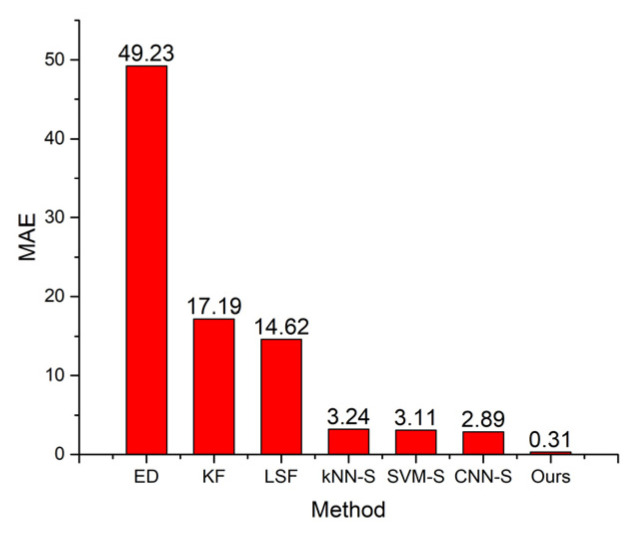
MAE versus different methods.

**Figure 10:**
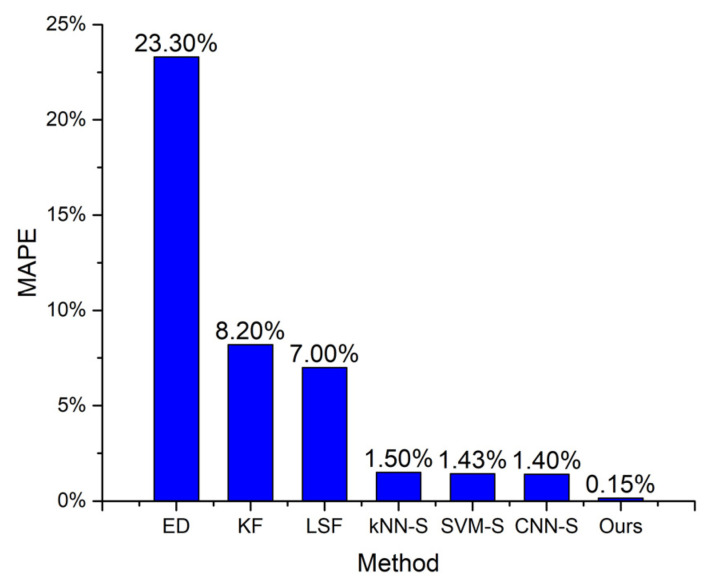
MAPE versus different methods.

The last three methods are similar to ours except that different classifiers are used. The results are given in Figure 9 and Figure 10. ED undoubtedly causes the most significant deviation from the actual distance among all the methods. The raw trace data contains positioning errors, making ED significantly overestimate the distance, especially when the walker is stationary. The outlier points also make the estimated distance larger than the actual distance. Its MAE reaches 49.23 m, and MAPE reaches 23.3%. 

KF and LSF can be categorized as the same class of method, which first removes the positioning errors from the raw trace while retaining the actual movement patterns, and then estimates the distance from the processed trace. These two methods can reduce the distance estimating error. The MAE values of KF and LSF decrease to 17.19 and 14.62 m, respectively. The MAPE values of KF and LSF decrease to 8.2% and 7%, respectively. However, a walker’s trace usually contains multiple parts that correspond to different movement statues. KF and LSF cannot perform very well in all these statuses. Therefore, these two methods still cause notable errors.

kNN-S, SVM-S, CNN-S, and our method are all status-based distance estimating methods. These methods divide the raw trace into multiple segments, use a specific classifier to determine each segment’s movement status, and apply different distance estimating methods to the segments according to their movement statuses. The distance overestimation caused by positioning errors when the moving target is stationary is eliminated. Using LSF to process the segments corresponding to the short-range intentional movement status significantly reduces the distance underestimation compared with KF. The MAE values of these four methods are below 3.3 m, and the MAPE values of these four methods are below 2%. 

Our method achieves the lowest distance error because the encoder–decoder classifier has the highest precision and recall rate. This proves that a status-based method can efficiently improve the distance estimation accuracy, and the underlying classifier is the key to achieving better performance.

## 5. Conclusions and Future Directions

This paper presents a movement status recognition-based distance estimation method to improve estimation accuracy. We divide the positioning traces into segments and use an encoder–decoder-based deep learning model to determine the motion status of each segment. Then, the distances of these segments are calculated by different distance estimation methods based on their movement statuses. 

Our method divides the raw trace into multiple segments containing an equal number of positioning points. This number is set manually to ensure the divided segment is homogeneous in its movement status characteristics. This may result in short segments that have negative effects on classifying performance. We will study adaptive segmentation methods with flexible time interval settings in future work. We will also consider integrating segmentation and classification to further improve the accuracy.

## Figures and Tables

**Table 1:** Summary of performance evaluation results.

	Precision	Recall	F1-Score
kNN	83.53%	82.69%	83.11%
SVM	86.13%	85.75%	85.94%
CNN	88.05%	87.76%	87.90%
Our classifier	97.81%	97.78%	97.79%

**Table 2:** Comparison of the execution time of different classifiers.

	Training Time	Average Classifying Time for One Segment
kNN	/	1.32 s
SVM	6.3 s	0.83 s
CNN	451 s	0.0079 s
Our classifier	876 s	0.0079 s

## Data Availability

The data used to support the findings of this study are available from the corresponding author upon request.
